# Effect of Hepatitis B Virus Infection on Sperm Quality and Outcomes of Assisted Reproductive Techniques in Infertile Males

**DOI:** 10.3389/fmed.2021.744350

**Published:** 2021-11-02

**Authors:** Zichun Wang, Wanpeng Liu, Mingming Zhang, Minglei Wang, Huaying Wu, Meisong Lu

**Affiliations:** Department of Reproductive, The First Affiliated Hospital of Harbin Medical University, Harbin, China

**Keywords:** male infertility, Hepatitis B virus, sperm, IVF, ICSI, IUI

## Abstract

**Background:** Hepatitis B virus (HBV) infection is one of the health problems and has adverse effects on public health. However, the consequences of male HBV carriers for assisted reproductive techniques (ART) remain unclear.

**Objective:** To examine whether men with HBV would impact sperm quality and the intrauterine insemination (IUI)/ *in vitro* fertilization (IVF)/ intracytoplasmic sperm injection (ICSI) outcomes.

**Methods:** We retrospectively analyzed data from 681 infertile couples for IUI/IVF/ICSI fresh cycle outcomes. Case group was 176 infertile couples with male HBV infection undergoing embryo transfer in our center (99 for IVF and 77 for ICSI) and 51 infertile couples for IUI. Negative control was 454 non-infected infertility couples, matched for female age, BMI and infertility duration (102 for IUI and 198 for IVF and 154 for ICSI).

**Results:** Sperm viability among infertile men with HBV infection was significantly lower than control group (74.1 ± 13.7 vs. 77.0 ± 12.8, *P* < 0.01). Sperm motility was significantly decreased in HBV positive men in comparison to the control group (32.5 ± 14.6 vs. 35.5 ± 12.9, *P* < 0.05). In IVF/ICSI cycles, two groups had similar results in two pronuclear (2PN) fertilization rate, implantation rate, clinical pregnant rate and abortion rate (*P* > 0.05). There was also no difference in the clinical pregnant rate and abortion rate in IUI cycles (*P* > 0.05).

**Conclusion:** Men with HBV infection will affect their sperm quality, but not affect the outcomes of ART.

## Introduction

Hepatitis B virus (HBV) is one of the major viruses threatening global public health of human, causing hepatic inflammation, cirrhosis and hepatocellular carcinoma in patients. According to the literature published in 2019, about 257 million people worldwide have been infected with HBV (1). Especially in China, it is estimated that 93 million people have been exposed to the HBV virus which cause the highest rate of HBV infection in the world (2).

HBV is a disease that is transmitted through fluids, many studies also showed that HBV DNA can be detected in urine, saliva, and other tissues beyond the liver and blood (3). HBV is not only able to pass through the blood-testis barrier and enter the sperm cell, but also integrate into their sperm chromosome. As early as 1985, Hadchouel et al. (4) noticed the presence of HBV DNA in seminal fluid from HBV patients, suggesting the possibility of vertical transmission of HBV to the offspring. Several studies have investigated the influence of HBV on sperm quality, however, the results are disparate (5–7). Nowadays, the number of HBV-infected men from infertile couples seeking assisted reproductive technology (ART) is increasing, including intrauterine insemination (IUI)/*in vitro* fertilization (IVF) and intracytoplasmic sperm injection (ICSI). It raised more concerns about the impact of HBV infection on ART outcome among these patients. But these reports on the *in-vivo* effects of HBV-infected men on IVF/ICSI outcomes are not yet conclusive (7–9). Especially, there are nearly no reports about the influence of HBV-infected men on IUI outcomes.

Our center has routinely screened the couples for antibodies for HBV, Hepatitis C virus (HCV), syphilis, and HIV (human immunodeficiency virus) prior to the first in assisted reproduction cycle. In view of the controversy, this retrospective study was conducted to examine the effects of HBV infection on sperm quality and ART treatment outcome.

## Materials and Methods

### Patients

The retrospective cohort study of 681 infertile couples from January 2016 to 2020 for the first IUI/IVF/ICSI cycle was undertaken in the Department of Reproductive, The First Affiliated Hospital of Harbin Medical University. Semen samples were obtained from the infertile male patients. Ethical approval was not required for this retrospective study. The HBV groups included a total of 51 IUI cycles and 99 IVF cycles and 77 ICSI cycles from HBsAg-seropositive husbands and HBsAg-seronegative wives. Control individuals were matched by BMI (±1), female age (±1 year), infertility duration (±1 year), and ART approach used (IUI or IVF or ICSI) and randomized in the ratio of 1:2 according to the ART treatment cycles. This was performed using a similar design to that previously described (10). Both husbands and wives in the control group were HBsAg-seronegative.

Infertile couples defined as the inability to achieve a clinically recognized pregnancy for at least 1 year of attempts—were met the inclusion criteria in the present study (11). Patients were excluded from this study if they met these criteria: chromosomal abnormalities, varicocele, long-term drug use, seropositive for HCV, and/or HIV, a history of surgery or congenital defects (urological or related to reproductive organs). Men with abnormal liver functional test (i.e., abnormal levels of aminotransferase, bilirubin and other coagulative parameters) were excluded in the study, either. None of the patients were diagnosed with acute hepatitis or received any antiviral treatment before assisted reproduction.

### Semen Analysis

Semen samples were collected by masturbation after 3–7 days of sexual abstinence and collected into sterile containers. All samples were analyzed after liquefaction for 30 min at 37°C. Volume, concentration, pH, concentration, motility and morphology were assessed according to WHO guidelines (World Health Organization, 1999). The analysis methods were described in the joint European Society of Human Reproduction and Embryology–Nordic Association for Andrology (ESHRE–NAFA) manual (12).

### ART Treatment Protocols

Ovulation was induced in all patients by either long or short protocol (13). Women initiated pituitary suppression in the mid-luteal phase with 0.1 mg/d GnRH agonist (GnRH-a; Triptorelin, Ferring GmbH, Germany) for 14–21 days (long protocol). When pituitary function was down-regulated (serum LH <5 IU/L, E2 <50 ng/L, endometrial thickness <4–5 mm), stimulation ovarian with 150–300 IU/d rFSH (Gonal F, Merk Serono, Switzerland) was administered. The short protocol involved pituitary down-regulation and ovulation induced from the 2nd to 4th day of the menstrual cycle by subcutaneous injection of GnRH-a and rFSH. Cycles were monitored by means of serial vaginal ultrasound scans and serum E2. As soon as 2–3 follicles of ≥18 mm were observed, final oocyte maturation was triggered by 5,000–10,000 IU of highly purified urinary human chorionic gonadotrophin (hCG; Livzon Bio-chemical Pharmaceutical Co., Zhuhai, China). Oocytes were collected by transvaginal ultrasound-guided needle aspiration ~36 h after hCG administration and mature oocytes were fertilized (IVF or ICSI) and cultured as described (14). One or two embryos were transferred on day 3 or 5 of the embryonic development. The patients in the IUI cycles were treated with appropriate ovulation induction on days 2–4 of the menstrual cycle. It entailed daily 2.5–5 mg letrozole (Fu Rui, Gudangdong Hengrui Medicine Co., Ltd.) for 5 days followed by a human menoposal gonadotrophin (HMG; Livzon Bio-chemical Pharmaceutical Co., Zhuhai, China) injection based on follicle size. Ten thousand IU hCG was administered when 1–2 follicles reached 18–20 mm in diameter. IUI was performed 24–36 h after the hCG injection (15).

### ART Outcome Measures

Women went back to the hospital 14 days after embryo transfer for the hCG pregnancy test, and an ultrasound scan was performed 14 days later in positive hCG test women to check the number, the site and the viability of gestational sacs and fetal hearts. Data on patients' characteristics and embryology were collected. These included patients' age, type, duration, and cause of infertility, ovarian reserve evaluation (cycle day 3 serum FSH), duration and total dose of gonadotropin treatment, sperm parameter, numbers of good-quality embryos, fertilization rate, implantation, pregnancy rates, and abortion rate. The implantation rate was defined as number of gestational sacs per embryo transferred. Clinical pregnancy rate was defined as the number of women with intrauterine gestational sacs per cycle with embryo transfer.

### Statistical Analysis

Statistical analyses were performed using software R version 3.2.4 (Lucent Technologies, Murray Hill, Kentucky, US). Categorical variables were compared between groups using Chi-squared tests. Two-sided *t*-tests were used to continuous variables (normally distributed data), while non-normally distributed measurement data were compared using the Wilcoxon rank sum test. Correlation-ship between serum viral load and sperm parameters was evaluated by the Spearman's rho. *P*-value < 0.05 was considered statistically significant.

## Results

In the present study, 247 HBsAg-seropositive participants were enrolled initially, out of which 20 men were later excluded for falling under the exclusion criteria such as treatment discontinuation, not performing the embryo transfer, or not providing the results of the pregnancy test. Finally, 227 HBsAg-seropositive men were included in the analysis. The seminal characteristics of 227 HBsAg-seropositive infertile men with 454 HBsAg-seronegative men as control group were shown in Table 1. Mean age of the patients with HBV infection and control group were 34.8 ± 4.9 and 34.7 ± 4.7 years old. Sperm concentration, volume and morphology were not significantly different between HBsAg-positive and HBsAg-negative men (*P* > 0.05). The sperm viability (74.1 ± 13.7 vs. 77.0 ± 12.8, *P* < 0.01) and progressive motility (32.5 ± 14.6 vs. 35.5 ± 12.9, *P* < 0.05) in HBV infection group was significantly decreased compared to the control group. To determine whether the viral HBV-DNA load could influence sperm parameters, the Spearman Correlation was calculated. No significant correlation was found between HBV-DNA load and sperm parameters (Supplementary Table S1).

**Table 1:** Seminal characteristics in HBV-positive men and negative controls.

	**HBV (+)**	**Control**	***P*-value**
*n*	227	454	
Male age (years)	34.8 ± 4.9	34.7 ± 4.7	NS^a^
Semen volume (ml)	2.7 ± 1.2	2.9 ± 1.2	NS^a^
Sperm concentration (10^6^/mL)	60.0 ± 31.9	64.6 ± 31.2	NS^a^
Sperm Viability (%)	74.1 ± 13.7	77.0 ± 12.8	**0.008** ^a^
Progressive motility (a+b) (%)	32.5 ± 14.6	35.5 ± 12.9	**0.017** ^b^
Normal sperm morphology (%)	9.7 ± 3.8	9.2 ± 3.9	NS^a^

*Values are presented as mean ± standard deviation*.

*P-values were obtained using ^a^Two-sided t-test or ^b^Wilcoxon rank sum test*.

*HBV, Hepatitis B virus; NS, not significant. Bold values means they are statistically significant*.

The data on clinical characteristics and outcomes of IVF are summarized in Table 2. There were no significant differences in the cycle characteristics between groups. The rates of fertilization, high-grade embryo, implantation, and clinical pregnancy were comparable between the two groups (*P* > 0.05). Though there was a trend of higher implantation rate, clinical pregnancy rate and abortion rate (42.8 vs. 35.9%; 58.5 vs. 53.5%; 17.2 vs. 16.9%, respectively, *P* > 0.05) among the HBV infection controls, the difference was not statistically significant.

**Table 2:** Ovarian stimulation variables and IVF results of HBV-positive and HBV-negative groups.

	**HBV group** ** (*n* = 99)**	**Control group** ** (*n* = 198)**	***P-*value**
Female age (years)	33.0 ± 4.0	33.0 ± 3.9	0.959^a^
Female BMI (kg/m^2^)	23.2 ± 3.4	23.3 ± 3.3	0.836^a^
Duration of subfertility (years)	4.4 ± 2.9	4.5 ± 3.0	0.847^a^
Primary infertility (*n*, %)	47/99 (47.4%)	104/198 (52.5%)	0.411^b^
Causes of subfertility (*n*, %)			
Male factor	11/99 (11.1%)	29/198 (14.6%)	0.400^b^
Tubal blockage	65/99 (65.6%)	113/198 (57.0%)	0.154^b^
Ovulation failure	19/99 (19.1%)	48/198 (24.2%)	0.326^b^
Endometriosis	4/99 (0.04%)	15/198 (0.07%)	0.240^b^
Unexplained	14/99 (14.1%)	34/198 (17.1%)	0.503^b^
Total dose of gonadotropin used (IU)	1,941.5 ± 744.2	1,993.3 ± 768.0	0.576^a^
Duration of gonadotropin stimulation (d)	9.7 ± 3.1	9.8 ± 2.9	0.808^a^
Serum E_2_ level on the day of hCG injection (pg/ml)	2,377.8 ± 1,323.9	2,469.1 ± 1,319.0	0.575^a^
Serum P level on the day of hCG injection (ng/ml)	0.7 ± 0.3	0.7 ± 0.3	0.988^a^
Endometrial thickness on the day of hCG injection (mm)	10.3 ± 2.2	10.5 ± 2.0	0.434^a^
2PN fertilization rate (%)	490/745 (65.7%)	1,030/1,516 (67.9%)	0.301^b^
High-grade embryo rate (%)	222/447 (49.6%)	468/894 (52.3%)	0.353^b^
Implantation rate (%)	81/189 (42.8%)	134/373 (35.9%)	0.110^b^
Clinical pregnancy rate (*n*, %)	58/99 (58.5%)	106/198 (53.5%)	0.409^b^
Abortion rate (*n*, %)	10/58 (17.2%)	18/106 (16.9%)	0.966^b^

*Values are presented as mean ± standard deviation*.

*P-values were obtained using ^a^Two-sided t-test or ^b^Chi-squared test*.

*HBV, Hepatitis B virus; BMI, body mass index; E_2_, estradiol; P, progesterone; hCG, human chorionic gonadotrophin; 2PN, two-pronuclear*.

To examine the clinical characteristics and outcomes of ICSI, we compared the differences between HBV-positive and HBV-negative groups, and no significant difference was observed (*P* > 0.05). Although there was a trend toward lower clinical pregnancy rate (49.3 vs. 50.0%, *P* > 0.05) and higher abortion rate (21.0 vs. 16.9%, *P* > 0.05) in the husband HBV-positive group, no statistical significance was reached (Table 3).

**Table 3:** Ovarian stimulation variables and ICSI results of HBV-positive and HBV-negative groups.

	**HBV group** ** (*n* = 77)**	**Control group** ** (*n* = 154)**	***P-*value**
Female age (years)	33.5 ± 5.0	33.5 ± 4.6	0.969^a^
Female BMI (kg/m^2^)	23.2 ± 3.0	22.6 ± 2.8	0.172^a^
Duration of subfertility (years)	5.9 ± 4.0	5.8 ± 3.7	0.794^a^
Primary infertility (*n*, %)	55/77 (71.4%)	96/154 (62.3%)	0.171^b^
Causes of subfertility (*n*, %)			
Male factor	56/77 (72.7%)	111/154 (72.0%)	0.917^b^
Tubal blockage	22/77 (28.5%)	47/154 (30.5%)	0.760^b^
Ovulation failure	14/77 (18.1%)	26/154 (16.8%)	0.805^b^
Endometriosis	4/77 (0.05%)	6/154 (0.03%)	0.647^b^
Unexplained	5/77 (0.06%)	10/154 (0.06%)	1.000^b^
Total dose of gonadotropin used (IU)	2,021.5 ± 802.5	2,054.0 ± 737.0	0.766^a^
Duration of gonadotropin stimulation (d)	10.2 ± 3.2	9.9 ± 2.5	0.121^c^
Serum E_2_ level on the day of hCG injection (pg/ml)	2,747.2 ± 1,278.0	2,539.6 ± 1,285.5	0.247^a^
Serum P level on the day of hCG injection (ng/ml)	0.7 ± 0.3	0.7 ± 0.5	0.331^c^
Endometrial thickness on the day of hCG injection (mm)	10.2 ± 1.7	9.9 ± 1.8	0.162^a^
2PN fertilization rate (%)	416/512 (81.2%)	814/1,020 (79.8%)	0.502^b^
High-grade embryo rate (%)	203/353 (57.5%)	398/675 (58.9%)	0.652^b^
Implantation rate (%)	48/143 (33.5%)	97/296 (32.7%)	0.867^b^
Clinical pregnancy rate (*n*, %)	38/77 (49.3%)	77/154 (50.0%)	0.925^b^
Abortion rate (*n*, %)	8/38 (21.0%)	13/77(16.8%)	0.586^b^

*Values are presented as mean ± standard deviation*.

*P-values were obtained using ^a^Two-sided t-test, ^b^Chi-squared test, or ^c^Wilcoxon rank sum test*.

*HBV, Hepatitis B virus; BMI, body mass index; E_2_, estradiol; P, progesterone; hCG, human chorionic gonadotrophin; 2PN, two-pronuclear*.

In IUI cycles, the mean age, Body Mass Index (BMI), infertility years and the mean FSH of the women in the two groups were compared, and the results showed that these differences were not statistically significant in the two groups (Table 4). The sperm concentration before and after sperm processing had no significant difference between the two groups. Sperm motility was also examined and the results showed that before sperm processing there was a significant difference between the two groups, that is, the husband HBV-negative group have better progressive motility (50.0 ± 31.6 vs. 69.2 ± 47.1, *P* < 0.01). After preparation, the progressive motile sperm count was not significantly higher in the negative group (23.5 ± 14.4 vs. 27.8 ± 17.4, *P* > 0.05). In this study, the mean recovery rate in the case and control groups were 54.5 ± 35.5% and 45.2 ± 25.0%, respectively, without significant difference (*P* > 0.05). The clinical pregnancy rate were 19.6% in the positive group and 20.5% in the negative group (*P* > 0.05). The abortion rate were 10.0% in the positive group and 14.2% in the negative group (*P* > 0.05).

**Table 4:** Comparison of the baseline variables and IUI results of HBV-positive and HBV-negative groups.

	**HBV group** ** (*n* = 51)**	**Control group** ** (*n* = 102)**	***P-*value**
Female age (years)	32.2 ± 4.2	32.0 ± 4.0	0.847^a^
Female BMI (kg/m^2^)	22.3 ± 2.9	22.4 ± 3.1	0.837^a^
Duration of subfertility (years)	4.1 ± 2.7	3.8 ± 1.9	0.705^b^
Primary infertility (n, %)	33/51 (64.7%)	73/102 (71.5%)	0.385^c^
Cycle day-3 FSH (mIU/mL)	5.6 ± 1.6	5.6 ± 1.4	0.921^a^
**Before preparation**			
Sperm concentration (10^6^/mL)	62.8 ± 25.2	64.3 ± 22.1	0.722^a^
Total progressive motile sperm count (*10^6^)	50.0 ± 31.6	69.2 ± 47.1	**0.006** ^b^
**After preparation**			
Sperm concentration (10^6^/mL)	95.4 ± 53.0	106.8 ± 60.5	0.235^a^
Total progressive motile sperm count (*10^6^)	23.5 ± 14.4	27.8 ± 17.4	0.104^a^
Recovery rate (%)	54.5 ± 35.5	45.2 ± 25.0	0.092^b^
Clinical pregnancy rate (*n*, %)	10/51 (19.6%)	21/102 (20.5%)	0.886^c^
Abortion rate (*n*, %)	1/10 (10.0%)	3/21(14.2%)	0.739^c^

*Values are presented as mean ± standard deviation*.

*P-values were obtained using ^a^Two-sided t-test, ^b^Wilcoxon rank sum test, or ^c^Chi-squared test*.

*HBV, Hepatitis B virus; BMI, body mass index; FSH, Follicle stimulating hormone. Bold values means they are statistically significant*.

## Discussion

Previous work has clearly demonstrated HBV infection is associated with a reduced motility and a higher proportion of apoptotic and necrotic sperm, resulting in lower fertility index (16). Furthermore, HBV infection could induce sperm chromosome aberrations and damage sperm genetic material (DNA) and mitochondrial membrane potential (17–19). Regarding the impact of HBV on sperm quality parameters, it is well-known that HBV infection may cause male infertility by impairing sperm function.

In this study, we found that HBV infected men had decreased sperm viability and progressive sperm motility in comparison to the control group. Both the values of sperm viability in HBV-positive and HBV-negative men were within the normal range, but the value in the HBV-negative group was much higher than the HBV-positive group (*p* < 0.01). Although both the mean of progressive sperm motility in the case and control group were below the normal reference v+ alues, the value in HBV infection was lower than control group (*p* < 0.05). The results of this study showed that HBV infection have negative impacts on sperm parameters.

These findings were similar to the previous studies that reported the effects of HBV infection on sperm parameters. Qian et al. (20) found the sperm parameters of infertile males with HBV infection were significantly lower than those of infertile males without infection and of normal males. Lorusso et al. (6) also found that sperm concentration, motility, viability, and morphology were significantly decreased in HBV-seropositive patients. In contrast, Oger et al. showed normal morphology in HBV-positive men compared to the negative group. Similarly, we observed the HBV-positive men had decreased sperm motility and viability but normal morphology compared to the controls (10). Other studies have demonstrated Hepatitis B virus S protein induced a loss of sperm membrane integrity in a dose-dependent manner (17, 21). And in our study, no significant correlation was found on semen quality and HBV-DNA load, although the viral HBV-DNA load in serum is varied widely in HBV-infected patients. It is similar to the study reported by Vicari et al. (22) who comparing the sperm parameters between HCV- and HBV-seropositive males. In view of these results, it is possible to conclude that HBV had adverse effects on sperm morphology might in a dose-dependent manner. Therefore, the molecular mechanism of HBV-induced impairment in human sperm needs to be further explored in the future.

HBV DNA could be integrated not only into the host hepatocytes, but also into the sperm chromosomes of HBV patients and induce chromosomal aberrations. It suggested that HBV infection might transmit vertically *via* the germ line to the next generation (18). When human sperm-mediated HBV genes were delivered into zona-free hamster oocytes *via* the IVF method, HBV genes were able to replicate and express in early embryonic cells (23). Actually, the sperm washing procedures could eliminate the presence of viruses, but the risk of HBV DNA integrated into sperm chromosome could not be eliminated (24). These results suggest that sperm cells of HBV patients might act as vectors for transmitting the HBV genes during IVF and ICSI procedures.

The effect of HBV-infected men on the outcomes in ART treatment cycle remain controversial. This study included two groups, HBV couples in which only the male partner was infected vs. control couples. Our results showed that no difference in fertilization, cleavage rates, clinical pregnancy rate, and abortion rate between infected males and controls, no matter ICSI cycles or IVF cycles. The results contradict previous studies in which couples whose male partner were infected have lower fertilization, implantation and pregnancy rates after IVF (8, 10). However, our results were in coincidence with Zhou et al. (7) and Lee et al. (25), they reported no adverse effect of HBV infection on IVF outcomes. Bu et al. (26) thought that male HBV infection has little impact on IVF outcomes. Our study also demonstrates that couples, in which male partners have infection with HBV, have similarly rates of 2PN fertilization, high-grade embryos acquisition, implantation, clinical pregnancy and early abortion in ICSI cycles compared to normal couples. Interestingly, Zhou et al. (7) reported ICSI could aggravate HBV transmission into the oocyte, and HBV-infected men had lower rates of 2PN fertilization (70.9 vs. 74.0%), high-grade embryos acquisition (57.6 vs. 60.4%), implantation (18.3 vs. 24.2%). and clinical pregnancy (31.2 vs. 39.3%) in ICSI cycles. However, Lutgens et al. (24) thought sperm washing could effectively reduce the risk of vertical transmission and prevent introduction of HBV into the oocyte in the case of ICSI. As previous studies found that hepatotropism was a prominent feature of HBV infection, the HBV DNA level in semen was lower compared with that in serum (27). In addition, these patients had been in a convalescent stage for more than 6 months, which exceed the spermatogenic cycle. According to two similar studies, they found that the rates of HBV positive embryos were only 16.6 and 21.3% in male HBsAg-positive/female HBsAg-negative couples, respectively (28, 29). It can assume that HBV DNA integrate into human sperm chromosomes, causing adverse effects on human sperm function, but that those infected embryos could not be fully functional, and the HBV positive males still have the opportunity to get uninfected sperm and embryos to fertilize and implant. These may explain why no adverse effect on outcomes of ICSI/IVF cycles was observed in the HBV-infected group. There is little information about HBV positive males of IUI cycles. In our research, the significant difference in total motile sperm count before sperm preparation (50.0 ± 31.6 vs. 69.2 ± 47.1, *P* < 0.01) disappeared after preparation (23.5 ± 14.4 vs. 27.8 ± 17.4, *P* > 0.05). And the two groups showed similar clinical pregnancy rate and abortion rate. It showed that the use of semen processing was effective enough to improve the properties of the sperm, leading to a high chance of fertility. Therefore, IUI might be beneficial for improving HBV-infected males fertility outcomes. However, these findings are insufficient to make a definite conclusion on the subject.

Semen quality and ART outcomes of HBV-infected men were examined in the present study, but the underlying molecular pathogenic mechanisms in human still remain to be investigated, especially whether the exogenous HBV-DNA fragment have a long-term effect on human gene mutation in assisted reproduction or not. On the other hand, one weakness of our current report is the absence results of abnormal liver functional test, which is restricted by our enrolled criteria and rigid procedure, so we could not know whether abnormal liver function could influence semen quality. These interpretations prompted the need for a prospective study consisting of larger sample.

In conclusion, this study confirms that HBV-seropositive has a negative impact on sperm viability and progressive motility of men. However, there was no significant difference in ART outcomes of the couples with husbands that were HBV infection.

## Data Availability Statement

The raw data supporting the conclusions of this article will be made available by the authors, without undue reservation.

## Ethics Statement

Ethical review and approval was not required for the study on human participants in accordance with the local legislation and institutional requirements. Written informed consent for participation was not required for this study in accordance with the national legislation and the institutional requirements.

## Author Contributions

ZW and WL performed the data analysis and drafted the manuscript. MZ participated in the data acquisition and interpretation. MW and HW contributed to the data analysis. ML designed the study. All authors reviewed and approved the manuscript.

## Conflict of Interest

The authors declare that the research was conducted in the absence of any commercial or financial relationships that could be construed as a potential conflict of interest.

## Publisher's Note

All claims expressed in this article are solely those of the authors and do not necessarily represent those of their affiliated organizations, or those of the publisher, the editors and the reviewers. Any product that may be evaluated in this article, or claim that may be made by its manufacturer, is not guaranteed or endorsed by the publisher.
