# Supplementary material for: Effect of Hepatitis B Virus Infection on Sperm Quality and Outcomes of Assisted Reproductive Techniques in Infertile Males
Source: Front Med (Lausanne). 2021 Nov 2;8:744350. doi: 10.3389/fmed.2021.744350 (PMC8592897; doi:10.3389/fmed.2021.744350)
Supplement: Supplementary file 1 [file Table_1.DOCX]

Table S1. Correlation between HBV-DNA load and seminal characteristics

| Variables | R value | P value |
| --- | --- | --- |
| Semen volume | 0.09387 | 0.15862 |
| Sperm concentration | 0.11402 | 0.08652 |
| Sperm Viability | 0.05843 | 0.38089 |
| Progressive motility (a+b) | 0.08156 | 0.22091 |
| Normal sperm morphology | -0.03660 | 0.59346 |

HBV, hepatitis B virus.
